# Tuberculous prostatitis: mimicking a cancer

**DOI:** 10.11604/pamj.2016.25.130.7577

**Published:** 2016-11-02

**Authors:** El Majdoub Aziz, Khallouk Abdelhak, Farih Moulay Hassan

**Affiliations:** 1 Department of Urology, Hassan II Hospital University Center, Fez, Morocco

**Keywords:** Genitourinary tuberculosis, prostate, cancer, Transrectal needle biopsy of the prostate

## Abstract

Genitourinary tuberculosis is a common type of extra-pulmonary tuberculosis . The kidneys, ureter, bladder or genital organs are usually involved. Tuberculosis of the prostate has mainly been described in immune-compromised patients. However, it can exceptionally be found as an isolated lesion in immune-competent patients. Tuberculosis of the prostate may be difficult to differentiate from carcinoma of the prostate and the chronic prostatitis when the prostate is hard and nodular on digital rectal examination and the urine is negative for tuberculosis bacilli. In many cases, a diagnosis of tuberculous prostatitis is made by the pathologist, or the disease is found incidentally after transurethral resection. Therefore, suspicion of tuberculous prostatitis requires a confirmatory biopsy of the prostate. We report the case of 60-year-old man who presented a low urinary tract syndrome. After clinical and biological examination, and imaging, prostate cancer was highly suspected. Transrectal needle biopsy of the prostate was performed and histological examination showed tuberculosis lesions.

## Introduction

Tuberculous prostatitis has mainly been described in immune-compromised patients. However, it can exceptionally be found as an isolated lesion in immune-competent patients. Tuberculosis involving the prostate gland, apart from being rare, can also mimic cancer of the prostate as well as chronic tuberculosis and therefore requires a high index of suspicion. We report a case of prostatic tuberculosis in a 60-year-old man, healthy and immune-competent patient. After clinical and biological examination, and imaging, prostate cancer was highly suspected. Trans-rectal needle biopsy of the prostate was performed and histological examination showed tuberculosis lesions.

## Patient and observation

**Figure 1:**
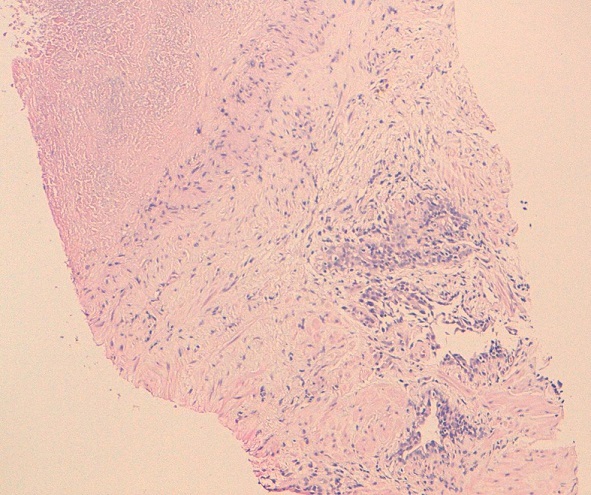
Histology: prostatic parenchyma seat beaches caseous necrosis that is surrounded by a band of epithelioid cells

**Figure 2:**
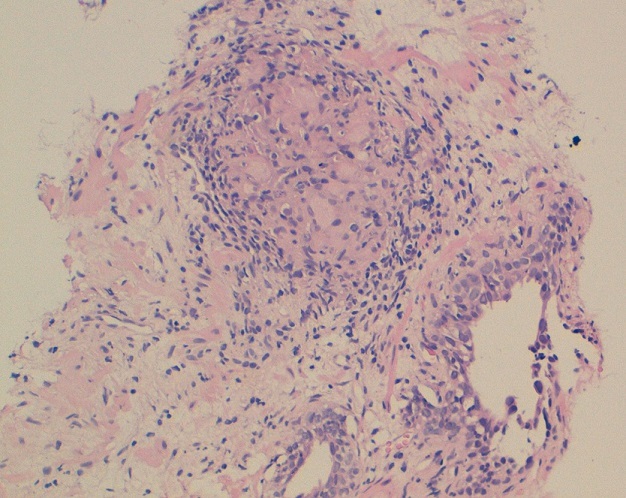
Histology: prostatic parenchyma seat granuloma epithelioid and giant cell

60-year-old man, lower socio-economic level, consulted for an obstructive lower urinary tract involving urinary frequency and dysuria lasting for about six months. These symptoms were accompanied by a low-grade fever Vesper, anorexia, asthenia and a weight loss (5kg in six months). The patient was vaccinated with BCG in childhood and he had no personal or family history of tuberculosis. Rectal touch showed an enlarged prostate with hard consistency and nodular surface. Biology found an elevation rate of prostatic specific antigen (PSA) (8ng/ml). The urine culture was sterile. HIV serology was negative. The pulmonary radiography was normal. The prostate ultrasound showed a heterogeneous prostate, enlarged, and whose weight was estimated at 35g. A prostate cancer was suspected, given the characteristics of clinical and ultrasound of the prostate gland and PSA elevation. A prostatic trans-rectal biopsy was performed. Histology disproved the existence of any neoplasic formation and there were more follicles with giant cells and caseous necrotic characteristic of prostate tuberculosis (Figure 1, Figure 2). We performed an intravenous urographic examination without finding any abnormalities in other structures of the urinary tract. The patient had received antibiotic treatment for six months with an intensive two-month four-drug tuberculosis (rifampicin, isoniazid, pyrazinamide and ethambutol) followed by a continuation phase of four months involving two tuberculosis drugs (isoniazid and rifampicin). Rapid sedation of symptoms, from the fourth week after initiation of specific treatment, was observed. After one year, the outcome was favorable with disappearance of voiding dysfunction and a normal PSA level.

## Discussion

The Genitourinary tuberculosis represents 10-14% of all locations of extra-pulmonary tuberculosis [1]. Prostate localization, especially if it is isolated, is rare [2, 3]. It's first described in 1882 by Jasmin [2]. Its incidence is estimated at 6.6% of the urogenital tuberculosis according Scotch Brady Urological Institute in Baltimore [2]. The prostatic achievement is often secondary to tuberculosis of the upper urinary tract [2, 3]. But it can also be primary or secondary to epididymal tuberculosis or bladder [3]. The hematogenous spreading is one of the common way contaminations. This infection is promoted by some immunosuppressive diseases in developed countries such as AIDS and taking of corticosteroid and chemotherapy. The risk of contamination prostate during intra-vesical instillation of BCG was also mentioned by some authors [2].

The clinical signs of lower urinary tract obstruction such as dysuria, urinary frequency and perineal heaviness can be observed. Digital rectal examination can enjoy enlarged prostate, elastic consistency, firm or stony or nodular as in our case [4]. Tuberculosis of the prostate may be difficult to differentiate from carcinoma of the prostate and the chronic prostatitis when the prostate is hard and nodular on digital rectal examination and the urine is negative for tuberculosis bacilli. In many cases, a diagnosis of tuberculous prostatitis is made by the pathologist, or the disease is found incidentally after trans-urethral resection. K. Huang et al. in Taiwan conducted a study on 10 patients over a period of 10 years, who all presented with digital rectal examination findings suggestive of Prostate cancer, but needle biopsy of the prostate revealed tuberculosis [5]. A. Kostakopoulos et al. also presented 5 cases of TB of the prostate, all of which were incidental histologic findings after Trans-urethral resection of the Prostate [6]. Tubercular serology by enzyme immunoassay (ELISA) or polymerase chain reaction (PCR) now allow a rapid laboratory diagnosis of tuberculosis with a sensitivity of respectively 80 and 95% [2], however these new tests are unfortunately not easily accessible in developing countries like ours. On the morphological examination, ultrasound is generally show enlarged prostate, heterogeneous structure with occasional areas of calcification and necrosis. The trans-rectal ultrasound provides sharper images and guide prostatic biopsy. Intravenous urography (IVU) was previously the examination of choice; this review, although no specific, can provide arguments strong presumption of genitourinary tuberculosis discovering a kidney chews, a cave or a small bladder.

In our case it performed to confirm the isolated localization of tuberculosis in the prostate. As for the scanner, it may reveal multiple lesions of low density reaching the different lobes of the prostate [7]. However, other non-tuberculous prostatic abscess may give similar aspects. The diagnosis is based on research of Mycobacterium tuberculosis (BK) in the urine or semen and / or histological examination of biopsy pieces. On histology, the macroscopic appearance depends on two opposing processes: one of destruction and caseation creating caves, another defense by limiting the extension of fibrosis lesions. It is this latter process that leads to obstructive phenomena. Prostatic lesions first take the appearance of yellowish streaks arranged in a “wheel spokes”. Thereafter, plates are formed caseous softening which leads to a true secondary prostatic abscess. Natural evolution can lead to the appearance of perineal fistulas [2, 8]. Involvement of the seminal vesicles is often associated with achieving prostate. The histological appearance is a typical granuloma with caseous necrosis and giant cells.

The treatment is primarily based on medical use of antibacillary drugs (isoniazid, rifampicin, pyrazinamide, streptomycin and ethambutol). Protocol, currently well codified, however, it can vary by country. Surgical treatment (endoscopic resection) found its directions in the case where there is an obstruction in the low urinary tract.

## Conclusion

Tuberculosis of the prostate may be difficult to differentiate from carcinoma of the prostate and the chronic prostatitis on digital rectal examination. Definitive diagnosis must be made by histopathological and bacteriologic studies. Transrectal ultrasound-guided needle biopsy of the prostate can yield a reliable diagnosis. Therefore, it is recommended as the method of choice for diagnosis and follow- up, as has been advocated for the diagnosis of prostate cancer.
